# IRF6 Is Involved in the Regulation of Cell Proliferation and Transformation in MCF10A Cells Downstream of Notch Signaling

**DOI:** 10.1371/journal.pone.0132757

**Published:** 2015-07-10

**Authors:** Talip Zengin, Burcu Ekinci, Cansu Kucukkose, Ozden Yalcin-Ozuysal

**Affiliations:** Department of Molecular Biology and Genetic, Izmir Institute of Technology, Izmir, Turkey; Wayne State University School of Medicine, UNITED STATES

## Abstract

IRF6, a member of Interferon Regulatory Factors (IRF) family, is involved in orofacial and epidermal development. In breast cancer cell lines ectopic expression of IRF6 reduces cell numbers suggesting a role as negative regulator of cell cycle. IRF6 is a direct target of canonical Notch signaling in keratinocyte differentiation. Notch is involved in luminal cell fate determination and stem cell regulation in the normal breast and is implicated as an oncogene in breast cancer. Notch activation is sufficient to induce proliferation and transformation in non-tumorigenic breast epithelial cell line, MCF10A. ΔNp63, which is downregulated by Notch activation in the breast, regulates IRF6 expression in keratinocytes. In this report, we investigate Notch-IRF6 and ΔNp63-IRF6 interactions in MCF10A and MDA MB 231 cells. We observed that in these cells, IRF6 expression is partially regulated by canonical Notch signaling and ΔNp63 downregulation. Furthermore, we demonstrate that IRF6 abrogation impairs Notch-induced proliferation and transformation in MCF10A cells. Thus, we confirm the previous findings by showing a tissue independent regulation of IRF6 by Notch signaling, and extend them by proposing a context dependent role for IRF6, which acts as a positive regulator of proliferation and transformation in MCF10A cells downstream of Notch signaling.

## Introduction

IRF6 is a transcription factor that belongs to the interferon regulatory factors (IRF) family, which is mainly involved in the regulation of immune response [1]. IRF6, on the other hand, has not been associated with the immunity, but was shown to be a major player in orofacial and epidermal development [2]. IRF6 mutations were initially identified in human congenital disorders that are characterized by cleft lip and palate [3]. Mice null for IRF6 [4] or carrying mutation in DNA binding domain [5] exhibited craniofacial developmental abnormalities and hyperproliferative epidermis that failed to terminally differentiate. In the breast, IRF6 was initially shown to directly interact with maspin, a tumor suppressor, in an immortalized normal mammary epithelial cell line, 1436N1, and have a decreased expression in invasive breast cancer cell lines and breast tumors [6]. Later, IRF6 was implicated as a negative regulator of cell proliferation. Cell cycle arrest resulted in IRF6 accumulation in MCF10A cells, non-tumorigenic immortalized breast epithelial cell line, while ectopic expression with adenoviral vectors in breast cancer cell lines MCF7 and MDA MB 231 led to decreased cell numbers [7].

Notch is an evolutionary conserved signaling pathway that controls a variety of cellular processes in development and tumorigenesis of several tissues. Upon binding of transmembrane ligands (Delta-like- 1 (DLL1), DLL3, DLL4, jagged1 (JAG1) and JAG2) to the Notch receptors (NOTCH1, -2, -3, -4) on the surface of neighboring cells, two sequential cleavages are induced that result in the release of notch intracellular domain (NICD). NICD translocates to the nucleus and converts the transcriptional repressor complex CSL (RBPjκ) into activator recruiting co-activators including mastermind-like-1 and initiates transcription of the target genes [8]. In the normal breast tissue, Notch signaling regulates luminal cell fate decision [9–11] and stem-cell self-renewal [12]. In the context of breast tumorigenesis, Notch signaling has been widely investigated since its first detection as an integration site for mouse mammary tumor virus, which results in constitutive expression of NICD and generation of mammary tumors [13, 14]. High expression levels of Notch receptors and ligands were found to be correlated with poor prognosis [15] while Numb, a negative regulator of Notch, was lost in a group of breast tumors [16, 17]. Functional analysis provided evidence that Notch activation is sufficient to transform the non-tumorigenic breast epithelial cell line MCF10A and required to maintain the transformed phenotype of breast cancer cell lines MCF7 and MDA MB 231 [17]. Notch signaling crosstalks with several developmental and oncogenic pathways including Wnt, Her2 and Ras [18], however its downstream mediators in breast tumorigenesis are not yet fully understood.

Like IRF6, mice mutant for Notch ligand JAG2 exhibited cleft palate phenotype indicating that the two molecules are involved in the regulation of similar developmental processes [19]. Analysis of transgenic mice carrying both IRF6 and JAG2 mutations later revealed that IRF6 and JAG2 signaling converge during palate adhesion but failed to show an interaction in terms of transcriptional regulation [20]. Recently, evidence was provided that Notch signaling and IRF6 directly interact in keratinocytes. It was shown that IRF6 is a direct Notch target gene that is induced during keratinocyte differentiation through the canonical, CSL-dependent, pathway. siRNA mediated knockdown of IRF6 counteracted Notch-induced differentiation and tumor suppression indicating that IRF6 is an essential mediator of Notch function in keratinocytes [21].

p63, similar to its homologs p53 and p73, is a transcription factor that has at least six different forms expressed from two different transcription start sites, each of which has three different variants at the C-terminal domain due to alternative splicing [22]. Similar to IRF6, p63 mutations were found in several human syndromes that exhibit cleft palate and lip formation [23] and p63 null mice had abnormal ectodermal development including undifferentiated epidermis [24, 25]. A direct link between ΔNp63, which has a shorter N-terminus that contains the transcriptional activation domain, and IRF6 has been established in keratinocytes by providing evidence that ΔNp63 binds to elements distal or proximal to IRF6 transcription start site and induces the expression of IRF6 [26, 27]. In return, IRF6 downregulates ΔNp63 via proteasome mediated degradation [27]. In the breast tissue, ΔNp63 is involved in epithelial cell fate decision and cell-matrix adhesion under the negative control of Notch signaling [11, 28].

Based on these findings, we investigated possible Notch-IRF6 and ΔNp63-IRF6 interactions in breast epithelial cells. We provide data demonstrating that Notch signaling positively regulates IRF6 expression, partially through canonical pathway, in breast epithelial cells. We show that downmodulation of IRF6 by shRNA impaired Notch-induced proliferation and transformation in MCF10A cells. Furthermore, we show that ΔNp63 downregulation may be involved IRF6 upregulation as an alternative mechanism to canonical Notch signaling. However, IRF6 silencing did not affect ΔNp63 expression suggesting a tissue-specific feedback mechanism that is functional in keratinocytes but not in breast epithelial cells.

Thus, in the present study, we confirm previous findings in keratinocytes by showing that IRF6 regulation by Notch signaling and ΔNp63 is not tissue specific and extend them by proposing a context dependent function for IRF6 in breast epithelial cells, in which it functions as a positive regulator of cell proliferation and transformation downstream of Notch.

## Materials and Methods

### Cell culture and viral infection

MCF10A and MDA MB 231 cells were obtained from ATCC. Dulbecco's Modified Eagle's Medium/Ham's F-12 Nutrient Mixture 1:1 growth medium including 25 mM HEPES (Hyclone) supplemented with 5% horse serum (Biological Industries), 20 ng/ml epidermal growth factor (Sigma), 500 ng/ml hydrocortisone (Sigma), 100 ng/ml cholera toxin (Sigma), and 10 μg/ml insulin (Sigma) was used to grow MCF10A cells. MDA MB 231 cells were grown in Dulbecco's Modified Eagle's Medium (Hyclone) supplemented with 10% fetal bovine serum (Biological Industries). All cell lines were maintained in a humidified incubator with 5% CO_2_ at 37°C.

Notch signaling was activated by overexpression of Notch1 intracellular domain using MSCV-NICD retrovirus [11]. Notch inhibition was managed either by overexpression of dominant negative form of Notch co-activator Mastermind-like 1 (DNMM) using MSCV-DNMM retrovirus or shRNA mediated silencing of canonical Notch mediator CSL using lentivirus. ΔNp63 was silenced by shRNA expressed from plko based lentivirus [11]. Empty MSCV retrovirus or lentivirus expressing shRNA against Green Fluorescent Protein (GFP) were used as control for each infection. Virus containing supernatants were prepared as described [11]. Cells were plated to 6-well plates at 250.000 cells/well density the day before infection and incubated with virus overnight. All assays and analyses were performed 48 hours after removal of the virus.

### RNA isolation and QRT-PCR

Total RNA was isolated using PureLink RNA Isolation Kit (Invitrogen), cDNA was synthesized by random hexamer primers using RevertAid First Strand cDNA Synthesis Kit (Fermentas). Semi-quantitative real-time RT-PCRs (QRT-PCR) was performed with Maxima SYBR Green/Fluorescein qPCR Master Mix (Fermentas) on an iCycler real-time PCR detection system (Bio Rad). Relative mRNA values represent the mean±S.D. of minimum of three independent experiments. All expression data were normalized to endogenous control gene TATA box-binding protein (TBP) expression. Each data was then normalized to control group within an experiment. Two-tailed Student’s t-test was used to calculate statistical significance. Following primer pairs were used: HEY1 5’-GGGAGGGGAACTATATTGAATTTT-3’, 5’-ATTTGTGAATTTGAGATCCGTGT-3’; HEY2 5’-AAGATGCTTCAGGCAACAGG-3’, 5’-GCACTCTCGGAATCCTATGC-3’; IRF6 5’-GCTCTCTCCCAATGACTGACCTGGA-3’, 5’-CCATGACGTCCAGCAGCTTGCTA-3’; TBP 5’-TAGAAGGCCTTGTGCTCACC-3’, 5’-TCTGCTCTGACTTTAGCACCTG-3’; ΔNp63 5’-ATGCCCAGACTCAATTTAGTGA-3’, 5’-TTCTGCGCGTGGTCTGTGT-3’.

### Protein preparation and immunoblotting

Cells were lysed in RIPA buffer and homogenized with 26G syringe. Total protein (20–100 μg) was subjected to SDS/PAGE followed by immunoblotting with rabbit polyclonal anti-IRF6 (1:400, ab58915, Abcam), and mouse monoclonal anti-p63 (1:200, sc8431, Santa Cruz). Mouse monoclonal anti-γ-tubulin (1:10000, T6557, Sigma) was used for equal loading control. Quantification of the western images were done by Gel Analysis tool of ImageJ [29]. All protein levels were normalized to tubulin, and then each data was normalized to the control group within the experiment. Two-tailed Student’s t-test was used to calculate statistical significance.

### Cell proliferation and viability assays

Cells were plated to 6-well plates at 250.000 cells/well density and the infection was done as explained above. 48 hours after infection, cells were incubated with 20 μM of BrdU for 4 hours followed by flow cytometry analysis using APC BrdU Flow Kit (BD Pharmingen) on FACSCanto (BD). An unstained control, in which the cells were not incubated with BrdU but treated with the APC BrdU Flow Kit along with the samples, was used to set up the gates. 10,000 cells excluding debris and doublets (R1) were analyzed for BrdU-APC signal. R2 gate, that had no positive events in unstained control, was set to analyze BrdU positive cell population. For MTT assay, 48 hours after infection, wells were incubated with 0.5mg/ml MTT (Amresco) for 4 hours followed by dissolving of tetrazolium salts in DMSO and colorimetric measurement at 570nm with a background subtraction at 650nm. All values were normalized to control group. BrdU positive cell percentages and MTT assay values represent the mean±S.D. of three independent experiments. Two-tailed Student’s t-test was used to calculate statistical significance.

### Soft agar assay

48 hours after infection, 3000 cells/well were added to 6-well plates in growth medium with 0.35% noble agar (BD Difco) on top of a solidified layer of 0.5% noble agar prepared in growth medium. Cells were fed with fresh growth medium twice a week for 8 weeks, after which colonies were stained with 0.005% crystal violet and analyzed under Olympus CKX41 microscope using Olympus DP25 camera and DP2-BSW application software. For each condition, five fields per well were analyzed. From each field, three images (each focused on a different layer) were taken. Only the colonies that were in the focus and bigger than 30 μm in diameter were counted. Total colony numbers were calculated from 15 images for each well. For each condition, average colony number per well, which was calculated from three independent experiments, was plotted. Colony diameter was calculated using all of the counted colonies for each condition. Colony number per well and diameter values represent the mean±S.D. of three independent experiments. Two-tailed Student’s t-test was used to calculate statistical significance.

## Results

### IRF6 expression is induced by Notch activation in MCF10A cells

**Fig 1:**
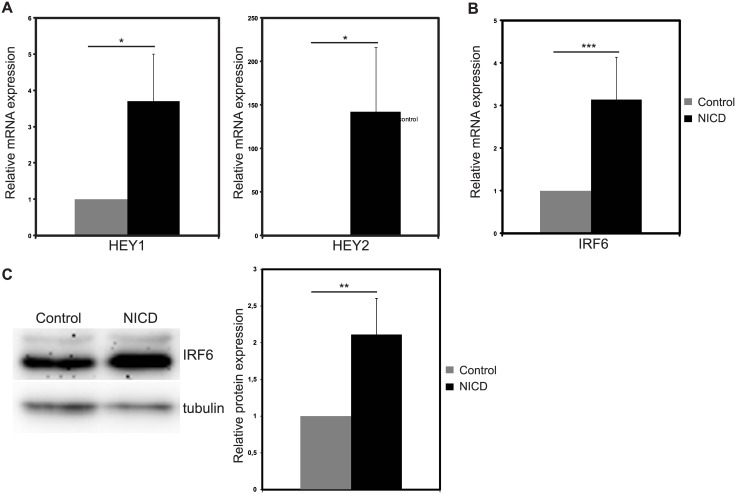
Notch activation induces IRF6 expression in MCF10A cells. Relative mRNA expression levels of (A) Notch target genes HEY1 and HEY2 and (B) IRF6 48 hours after infection with control (grey bars) or NICD expressing (black bars) retrovirus. (C) IRF6 protein levels 48 hours after infection with control or NICD expressing retrovirus (Left: representative western image, Right: Quantification of the protein bands). Values represent mean±S.D. of three independent experiments. (p values: *: <0.05, **: <0.02, ***: <0.003).

To assess whether Notch signaling regulates IRF6 expression in breast epithelial cells, we used immortalized, non-tumorigenic breast epithelial cell line with near diploid karyotype, MCF10A, which do not have detectable Notch activity [17, 30]. In MCF10A, we activated Notch signaling by ectopic expression of Notch1 intracellular domain (NICD), which is the active form of the receptor, via retroviral expression system. 48 hours after infection, mRNA levels of Notch target genes HEY1 and HEY2 were upregulated by 3.7- and 142-fold, respectively, in NICD infected cells compared to the cells infected with control virus, showing that Notch signaling was activated successfully (Fig 1A). Upon Notch activation, IRF6 mRNA was upregulated by 3.1-fold (Fig 1B) and a 2.1 fold increase in IRF6 protein was observed (Fig 1C). Thus, we provided evidence that IRF6 expression is induced by Notch activation in MCF10A cells.

### IRF6 is required for Notch-induced cell proliferation and transformation in MCF10A cells

**Fig 2:**
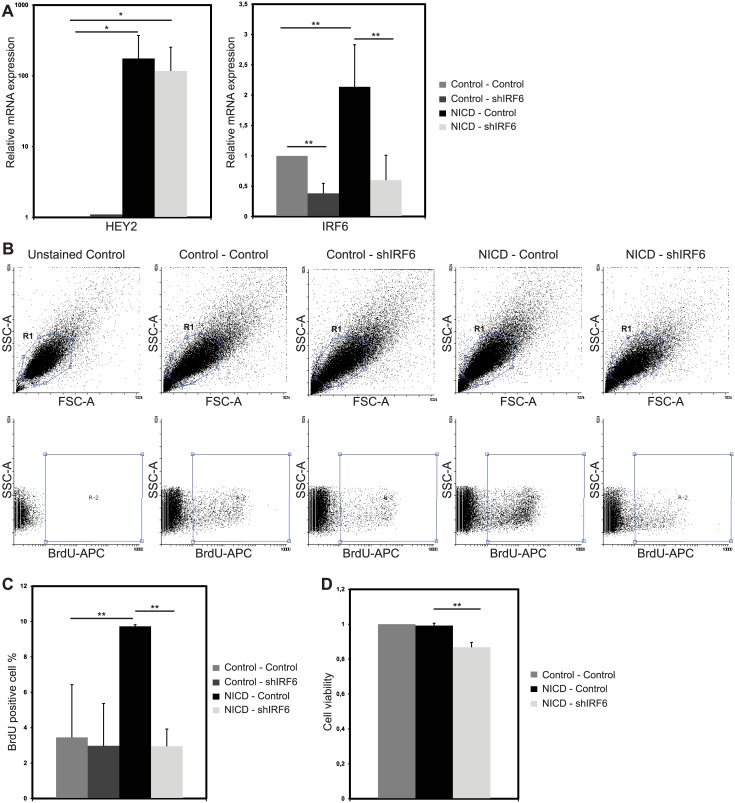
IRF6 is required for Notch induced cell proliferation. (A) Relative mRNA expression levels of Notch target gene HEY2 and of IRF6 in MCF10A cells infected with (i) two control viruses, (ii) control and IRF6 shRNA (shIRF6) expressing viruses, (iii) NICD and control shRNA expressing viruses or (iv) NICD and shIRF6 expressing viruses. (B) Representative dot plots of BrdU FACS analysis. R1 population (upper panel) were analyzed for BrdU-APC signal. R2 population (lower panel) shows events positive for BrdU-APC signal. (C) BrdU positive cell percentage in MCF10A cells, infected as indicated above, after 4 hours of BrdU incorporation. (D) MTT assay values indicating cell viability of MCF10A cells infected as indicated above. Values represent mean±S.D. of three independent experiments. (p values: *: < 0.05, **: <0.006).

Notch activation increases proliferation in MCF10A cells [17]. Since, IRF6 was shown to act as a negative regulator of cell proliferation in breast epithelial cells [7], we investigated whether its upregulation by Notch is involved in regulation of Notch-induced cell proliferation and transformation. For this purpose, we generated “NICD—Control” and “NICD—shIRF6” MCF10A cells by double infection. In both, Notch signaling was successfully activated upon infection with NICD virus as demonstrated by significant increase in Notch target gene HEY2 mRNA (Fig 2A). Then, we showed that NICD induced IRF6 upregulation was reduced by 70% in “NICD—shIRF6” group, which was also infected with virus encoding shRNA against IRF6 (shIRF6) compared to the “NICD—Control” group, which was infected with the control shRNA virus (Fig 2A).

Proliferation of the modulated cells was assessed by BrdU assay, in which MCF10A cells were incubated with BrdU for 4 hours and analyzed by flow cytometry 48 hours after infection. Notch activation increased the actively proliferating cell population, as expected [17], with an increase in BrdU positive population from 4% in “Control—Control” group to 10% in “NICD—Control” group (Fig 2C). When Notch cannot upregulate IRF6, the percentage of actively proliferating cells dropped to 3%, compared to “NICD—Control” group. Silencing of IRF6 only, did not affect BrdU incorporation rate (Fig 2C). Cell viability was assessed by MTT assay, in which the absorbance of formazan precipitate is correlated with the number of the alive cells [31]. In response to IRF6 abrogation cell viability was reduced by 15% in Notch activated cells (Fig 2D). Thus, IRF6 upregulation is required for Notch induced cell proliferation.

**Fig 3:**
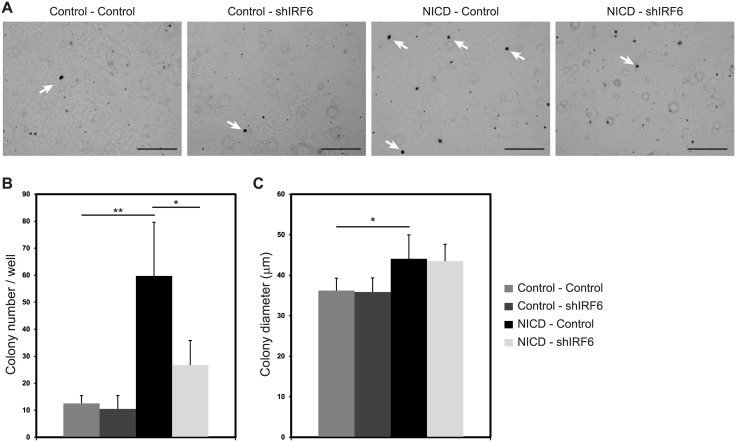
IRF6 is required for Notch induced transformation. (A) Photomicrographs of MCF10A cells infected with (i) two control viruses, (ii) control and IRF6 shRNA (shIRF6) expressing viruses, (iii) NICD and control shRNA expressing viruses or (iv) NICD and shIRF6 expressing viruses and grown in soft agar for 8 weeks. Arrows indicate representative colonies that are in focus and counted. Scale bar: 500 μm. Number of colonies (bigger than 30 μm in diameter) per well (B) and average colony size (C) are shown for each condition. Values represent mean±S.D. of three independent experiments. (p values: *: <0.03, **: <0.004).

MCF10A cells, which lack the ability to grow in anchorage independent way [30] are transformed by Notch activation, and form colonies in soft agar [17]. In order to test the role of IRF6 in Notch-induced transformation, MCF10A cells, modulated as explained above, were grown in soft agar for 8 weeks and number and size of the colonies were analyzed following crystal violet staining (Fig 3A). Notch activation increased average colony number per well from 13 in “Control—Control” group to 60 in “NICD—Control” group (Fig 3B). When IRF6 was silenced in Notch active cells, colony number was significantly reduced to 27 in “NICD—shIRF6” group (Fig 3B) indicating that IRF6 is required for Notch induced transformation of MCF10A cells. Colony size, on the other hand, was increased by Notch activation but not affected by IRF6 abrogation (Fig 3C), indicating that once transformed the number of cycles proliferating cells underwent through was independent of IRF6. Silencing of IRF6 only, did not affect neither number nor size of the colonies (Fig 3B and 3C).

Upon transformation, Notch active cells might have acquired alternative mechanisms to compensate the inhibitory effects of IRF6 loss. Proliferation analysis were done 48 hours after infection, however, transformation analysis were done 8 weeks after the infection, which gives the transformed cells time to activate alternative pathways to promote proliferation. The cells that acquired alternative mechanisms could have been selected over the others and compensate for the initial decrease in the proliferation rate.

Based on these results, it can be concluded that IRF6 is an essential mediator of Notch signaling in proliferation and transformation of MCF10A cells.

### Notch inhibition in breast cancer cell line MDA MB 231 reduces IRF6 expression

**Fig 4:**
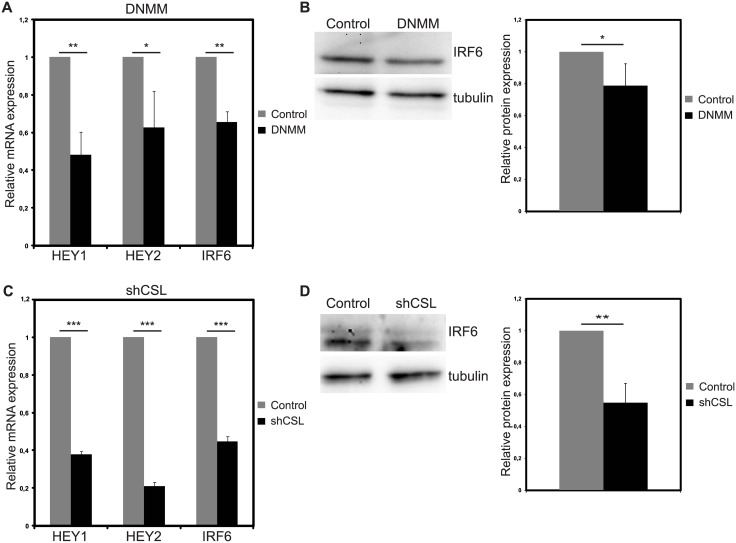
Notch inhibition reduces IRF6 expression in MDA MB 231 cells. Notch signaling was inhibited via overexpression of dominant negative mastermind (DNMM) (A and B) or silencing of Notch signaling mediator RBPjκ/CSL via shRNA (shCSL) (C and D). Relative mRNA expression levels of Notch target genes HEY1 and HEY2 and of IRF6 (A) and protein levels of IRF6 (B) 48 hours after infection with control (grey bars) or DNMM (black bars) expressing retrovirus (Left: representative western image, Right: Quantification of the protein bands). Relative mRNA expression levels of Notch target genes HEY1 and HEY2 and of IRF6 (C) and protein levels of IRF6 (D) 48 hours after infection with control (grey bars) or shCSL (black bars) expressing retrovirus (Left: representative western image, Right: Quantification of the protein bands). Values represent mean±S.D. of three independent experiments. (p values: *: <0.05, **: <0.004, ***: <0.0003).

To test whether Notch-IRF6 connection is also relevant in the context of endogenous tumorigenic Notch activation, we switched to a breast cancer cell line with high Notch activity, MDA MB 231 [17]. Notch signaling was inhibited by two different approaches; first, by overexpression of dominant negative form of Notch co-activator Mastermind-like 1 (DNMM) and second, by silencing the canonical Notch mediator CSL using shRNA. In DNMM overexpressing cells, HEY1 and HEY2 mRNA levels were reduced by 50% and 40%, respectively, compared to control infected cells indicating that Notch inhibition was achieved (Fig 4A). IRF6 mRNA was reduced by 35% in DNMM overexpressing cells compared to control infected cells (Fig 4A). 20% reduction in IRF6 protein could be observed in response to DNMM (Fig 4B). When specifically canonical Notch signaling was inhibited by shRNA against CSL (shCSL), IRF6 was downregulated by 55% at mRNA level (Fig 4C) and 45% at protein level (Fig 4D) compared to cells expressing control shRNA. 60% and 80% reduction in HEY1 and HEY2 mRNA expression in shCSL infected cells indicates that Notch signaling was successfully inhibited (Fig 4C). Overall, the data suggest that IRF6 expression in breast cancer cell lines with active Notch signaling is, at least partially, dependent on canonical Notch signaling.

### IRF6 upregulation is partially regulated by ΔNp63 downregulation

**Fig 5:**
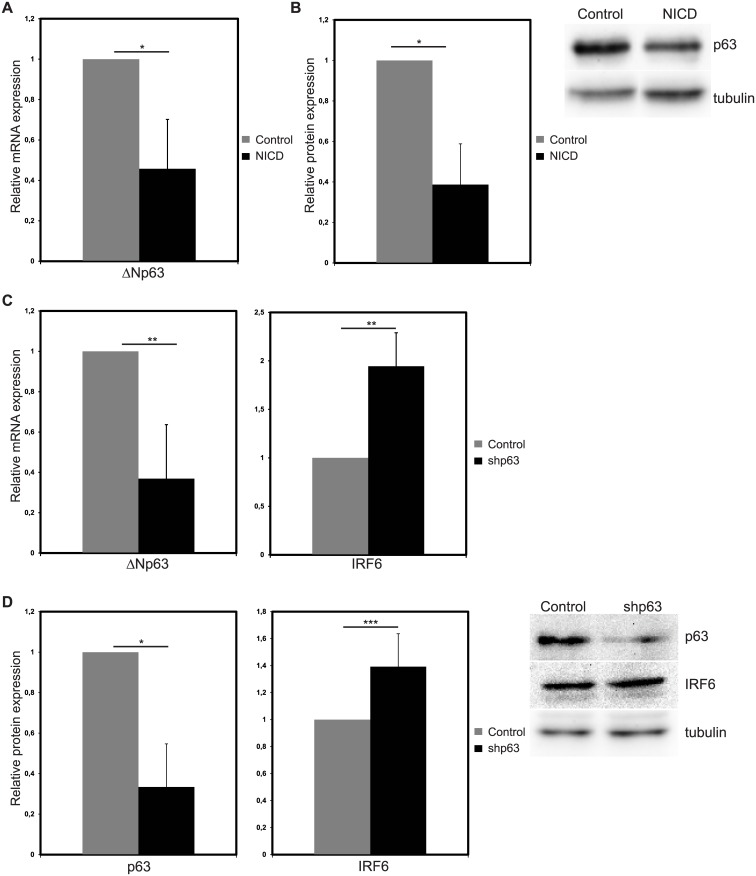
ΔNp63 silencing increases IRF6 expression. Relative mRNA (A) and protein (B) expression of ΔNp63 in MCF10A cells 48 hours after infection with control (grey bars) or NICD expressing (black bars) retrovirus (Left: Quantification of the protein bands, Right: representative western image). Relative mRNA (C) and protein (D) expression of ΔNp63 and IRF6 in MCF10A cells 48 hours after infection with lentivirus expressing control shRNA (grey bar) or shRNA against ΔNp63 (shp63) (black bars) (Left: Quantification of the protein bands, Right: representative western image). Values represent mean±S.D. of three independent experiments. (p values: *: <0.006, **: <0.02, ***: <0.05).

ΔNp63, which is negatively regulated by Notch signaling in the breast tissue [11, 28], controls IRF6 expression in keratinocytes [26, 27]. Thus, we wanted to assess whether ΔNp63 regulation is involved in Notch induced IRF6 expression in breast epithelial cells in addition to canonical Notch signaling. First, we ascertained that Notch induction downregulated the expression of ΔNp63, dominant p63 isoform in breast epithelial cells [11, 32], mRNA (Fig 5A) and protein (Fig 5B) under our experimental conditions, in MCF10A cells. Then, we used shRNA to downregulate ΔNp63 (shp63), which reduced mRNA expression by 60% (Fig 5C) and protein expression by 65% (Fig 5D) compared to the cells infected with control shRNA virus. Upon silencing of ΔNp63, IRF6 mRNA was increased by 1.9-fold (Fig 5C) and protein was upregulated by 1.4 fold (Fig 5D). Thus, p63 downregulation is sufficient to upregulate IRF6 expression, suggesting that Notch may induce IRF6 expression through downregulation of ΔNp63.

### ΔNp63 expression is not regulated by IRF6

**Fig 6:**
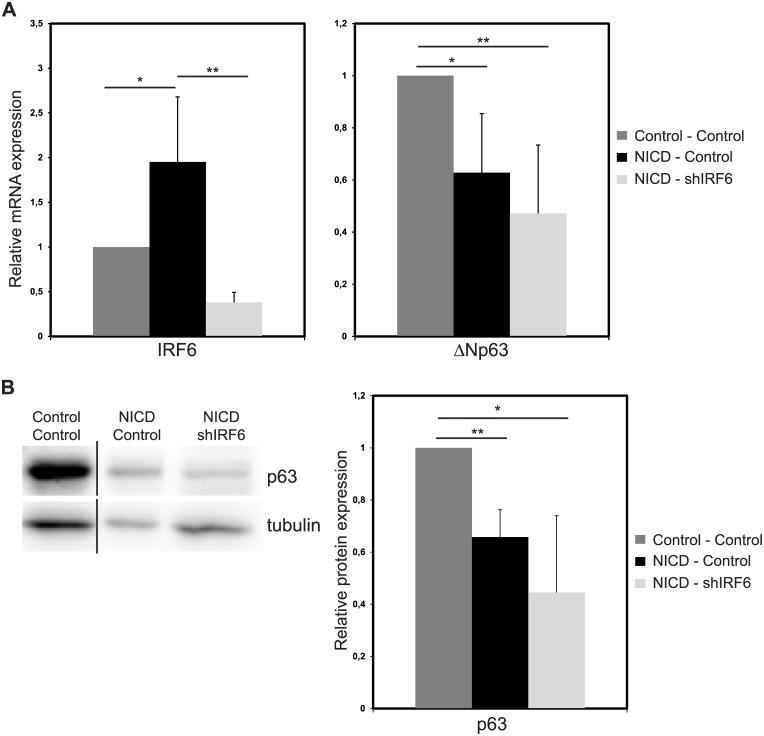
Notch induced downregulation of ΔNp63 is not mediated through IRF6. Relative mRNA expression levels of IRF6 and ΔNp63 (A) and protein expression of IRF6 (B) in MCF10A cells infected with (i) two control viruses (grey bars), (ii) NICD and control shRNA expressing viruses (black bars) or (iii) NICD and IRF6 shRNA (shIRF6) expressing viruses (light grey bars) (Left: representative western image, Right: Quantification of the protein bands). Values represent mean±S.D. of three independent experiments. (In panel B, first lane was loaded on the same gel into a well that is not adjacent to the other two wells.) (p values: *: <0.04, **: <0.007).

In keratinocytes, overexpression of IRF6 downregulated ΔNp63 via proteasome mediated degradation [27]. To test, whether the same mechanism works in breast epithelial cells, we used “NICD—Control” and “NICD—shIRF6” cells. In “NICD—Control” group IRF6 mRNA expression was increased by 1.9-fold while ΔNp63 mRNA was decreased by 40% (Fig 6A) compared to “Control- Control” group, showing that double viral infection did not interfere with the effects of Notch activation on IRF6 and ΔNp63 expression. In “NICD—shIRF6” condition, we managed to avert IRF6 upregulation by Notch as shown by 80% reduction in IRF6 mRNA (Fig 6A) compared to “NICD—Control” cells. ΔNp63 mRNA (Fig 6A) and protein (Fig 6B) expression remained downregulated in “NICD—shIRF6” cells and did not significantly change compared to “NICD—Control” group. Thus, we provided evidence that IRF6 upregulation by Notch is not involved in ΔNp63 regulation neither at mRNA nor at protein level in breast epithelial cells suggesting that this feedback mechanism is tissue specific.

## Discussion

Notch signaling and IRF6 have long been implicated in similar developmental processes like palate adhesion [5, 19] and keratinocyte differentiation [4, 5, 33]. However, only recently a direct interaction between the two genes has been revealed in keratinocytes showing that IRF6 is a direct target of canonical Notch signaling and is a mediator of its pro-differentiation and tumor suppressor function [21]. In line with the previous findings, we showed that IRF6 expression is regulated by Notch signaling in non-tumorigenic and tumorigenic breast epithelial cells indicating a tissue independent control mechanism. Our data suggest that the regulation is mediated by two different mechanisms; canonical Notch pathway and Notch induced ΔNp63 downregulation.

Upstream regulators of IRF6 are not well documented. In addition to recently described role of Notch; p63 and TGFβ signaling are known to directly regulate IRF6 expression. During palate development, TGFβ signaling induces IRF6 expression upon binding of SMAD4 to IRF6 promoter [34]. However, Notch signaling is known to repress TGFβ signaling in breast [35, 36], making it a less likely mechanism to explain Notch induced IRF6 expression. Yet, whether TGFβ regulates IRF6 expression in breast epithelial cells remains as an open question. ΔNp63 is known to be downregulated by Notch activation in breast epithelial cells [11, 28]. ΔNp63 regulates IRF6 expression by binding to elements distal or proximal to IRF6 transcription start site in keratinocytes [26, 27]. In contrast to positive regulation of IRF6 by ΔNp63 in keratinocytes, in this study, we provided evidence that shRNA mediated downregulation of ΔNp63 increased IRF6 expression. Thus, we propose an alternative model, in which Notch indirectly induces IRF6 expression by downmodulating ΔNp63. Yet, why the removal of the positive regulator, ΔNp63, increases target expression, IRF6, remains elusive. IRF6 positively regulates its own expression by binding to three IRF6 responsive elements, two in the promoter area and one in the distal region [37]. The binding site at the distal region exactly overlaps with the ΔNp63 binding site [37], raising the possibility of a competition between the two factors for binding to this site. In our system, removal of ΔNp63 upon Notch activation or shRNA mediated downregulation may shift the balance towards IRF6 binding and that in turn may induce its expression.

A reciprocal interaction was proposed between IRF6 and ΔNp63 in keratinocytes in the way of IRF6 induced proteasome mediated ΔNp63 degradation [27]. In MCF10A, we did not observe an effect of IRF6 depletion on ΔNp63 suggesting that Notch induced ΔNp63 downregulation is not mediated by IRF6. However, it should be noted that IRF6 induced ΔNp63 degradation was restricted to the differentiating keratinocytes and no effect was observed in proliferating cells [27]. Together with our findings, this points to a tissue and cell-type specific feedback mechanism between ΔNp63 and IRF6. In the normal breast tissue, IRF6 expression reaches to its maximum levels in lobuloalveolar cells during lactation suggesting that IRF6 may have a role in differentiation of breast epithelial cells [38]. We cannot ignore a scenario, where IRF6 regulates p63 in different types of breast epithelial cells, such as luminal or luminal progenitor, or at a different stage of differentiation, such as lactation. Thus, our current observation in MCF10A cells needs further investigation in different differentiation stages of breast epithelial cells.

IRF6 was implicated as a tumor suppressor in squamous cell carcinoma (SCC). IRF6 expression was downmodulated in SCC tumors, where its overexpression in SCC cell lines reduced colony formation, while its silencing induced matrigel invasion [21, 37]. In the breast, it was shown that IRF6 expression was reduced in breast cancer cell lines and invasive tumors [6]. Furthermore, IRF6 was accumulated upon cell cycle arrest in MCF10A cells and its adenoviral overexpression in breast cancer cell lines reduced cell numbers [6, 7], implicating IRF6 as a negative regulator of cell cycle. Here, we provided evidence that IRF6 might have an alternative role downstream of Notch signaling in breast. We showed that silencing of IRF6 impaired Notch-induced proliferation and transformation in MCF10A cells, suggesting a growth promoting role. Dual role of IRF6 may be context dependent, where in combination with other Notch targets IRF6 might be involved in a program to activate cell proliferation and transformation, while in the absence of Notch signaling its accumulation might simply prevent cell cycle progression. In line with a context dependent function, we showed that silencing of IRF6 in the absence of Notch activation did not affect proliferation or transformation of MCF10A cells. In parallel, keratinocytes isolated from wild-type and IRF6 knockout embryos showed no difference in BrdU incorporation or cell cycle profile in short-term culture [39]. Only in the long-term, 10–12 days in culture, IRF6 knockout cells reached to higher numbers [39], indicating that IRF6 alone is not enough to regulate cell proliferation but needs the proper context to be established.

IRF6 was shown to be phosphorylated, in response to mitogenic stimuli and subsequently targeted for proteasomal degradation [6, 7]. It was suggested that phosphorylated and unphosphorylated forms might have different functions. Phosphorylated IRF6 may facilitate exit from G0 and entry into G1 prior to degradation suggesting a growth-stimulating role, while unphosphorylated IRF6 accumulation may simply induce cell cycle arrest [7]. In our study, we failed to detect phosphorylated form of IRF6 neither upon treatment with phosphatase inhibitors nor in response to proteasome inhibition (data not shown). However, we cannot exclude the scenario, where Notch activation not only increases IRF6 expression but also induces its phosphorylation. Phosphorylated form might be the main form acting downstream of Notch signaling to induce its proliferation inducing functions. Thus, IRF6 downstream of Notch may be required for its growth promoting functions instead of steady state function that induces cell cycle arrest.

Downstream targets that regulate IRF6 functions are not well known. Recently, two groups revealed IRF6 targets in normal human keratinocytes and zebrafish periderm [37, 40]. Gene expression profiling and ChIP sequencing identified IRF6 upregulated genes involved in proliferation, angiogenesis, cell adhesion, and interaction with extracellular matrix [37]. Whether similar group of genes are also regulated by IRF6 in breast remains elusive. Identification of downstream targets will be the key to understand context dependent functions of IRF6 in the breast.
